# Development, characteristics and impact of quality improvement casebooks: a scoping review

**DOI:** 10.1186/s12961-021-00777-z

**Published:** 2021-09-08

**Authors:** Natalie N. Anderson, Anna R. Gagliardi

**Affiliations:** Toronto General Hospital Research Institute, University Health Network, Toronto, Canada

**Keywords:** Casebook, Quality improvement, Experiential knowledge, Implementation science, Knowledge translation, Scoping review

## Abstract

**Background:**

Quality improvement (QI) casebooks, compilations of QI experiences, are one way to share experiential knowledge that healthcare policy-makers, managers and professionals can adapt to their own contexts. However, QI casebook use, characteristics and impact are unknown. We aimed to synthesize published research on QI prevalence, development, characteristics and impact.

**Methods:**

We conducted a scoping review by searching MEDLINE, EMBASE, CINAHL and SCOPUS from inception to 4 February 2021. We extracted data on study characteristics and casebook definitions, development, characteristics (based on the WIDER [Workgroup for Intervention Development and Evaluation Research] framework) and impact. We reported findings using summary statistics, text and tables.

**Results:**

We screened 2999 unique items and included five articles published in Canada from 2011 to 2020 describing three studies. Casebooks focused on promoting positive weight-related conversations with children and parents, coordinating primary care-specialist cancer management, and showcasing QI strategies for cancer management. All defined casebooks similarly described real-world experiences of developing and implementing QI strategies that others could learn from, emulate or adapt. In all studies, casebook development was a multistep, iterative, interdisciplinary process that engages stakeholders in identifying, creating and reviewing content. While casebooks differed in QI topic, level of application and scope, cases featured common elements: setting or context, QI strategy details, impacts achieved, and additional tips for implementing strategies. Cases were described with a blend of text, graphics and tools. One study evaluated casebook impact, and found that it enhanced self-efficacy and use of techniques to improve clinical care. Although details about casebook development and characteristics were sparse, we created a template of casebook characteristics, which others can use as the basis for developing or evaluating casebooks.

**Conclusion:**

Future research is needed to optimize methods for developing casebooks and to evaluate their impact. One approach is to assess how the many QI casebooks available online were developed. Casebooks should be evaluated alone or in combination with other interventions that support QI on a range of outcomes.

**Supplementary Information:**

The online version contains supplementary material available at 10.1186/s12961-021-00777-z.

## Background

Quality improvement (QI) refers to the use of strategies that promote and support evidence-informed practice to enhance safety and effectiveness, and the likelihood of desirable health outcomes [1]. Guidance is available on the basic steps of QI [2], and there are many QI strategies to choose from, including clinician reminder systems, facilitated relay of clinical data to clinicians, audit and feedback, clinician education, patient education, promotion of self-management, patient reminders, organizational change approaches, and financial or regulatory incentives [3]. However, how to choose and implement a QI strategy for a given context remains unclear. Systematic reviews of the effectiveness of a range of QI strategies revealed that studies were methodologically biased and must be interpreted with caution; strategies differed across studies, limiting interpretation; and strategy impact was inconsistent, leaving uncertainty about their value [4–6]. Furthermore, numerous barriers at multiple levels can challenge improvements in professional practice and associated clinical outcomes [7].

Given the shortcomings of empirically generated evidence on the effectiveness and applicability of QI strategies, other forms of knowledge may be relevant to those undertaking QI. Experiential knowledge refers to dynamically created context-specific learning acquired through one’s own personal experience or through communication from others about their experience [8]. Interviews and focus groups with representatives of public health units revealed that experiential knowledge was used to inform programme planning decisions, including identifying the need, bringing a team together, and designing and developing the programme [9]. To generate insight on how QI practices are spread, Guzman et al. drew from the knowledge management and organizational learning literatures to develop a framework that identified three processes required to adopt QI practices: transfer of knowledge about practices between organizations, copying best practices, and translating them into a new context [10]. The framework also proposed that experiential knowledge about QI increases in relevance as organizational complexity increases. One way to share experiential QI knowledge is through QI collaboratives, which bring together groups of healthcare workers from different organizations to systematically improve one aspect of the quality of their services through joint learning and sharing of experiences [11]. In a systematic review of 64 studies of QI collaboratives, 83% reported improvements in measured outcomes; however, studies varied in settings, topics and populations, and many provided insufficient description of the collaboratives such that they could be replicated [12]. In-person or virtual coaching has also been used to transfer experiential knowledge and support QI with positive impact on QI knowledge and self-efficacy, decision-making, staff satisfaction and quality of clinical care [13, 14]. However, evidence is sparse on the optimal characteristics and roles of coaches, also referred to as knowledge brokers, opinion leaders, facilitators or change agents, or their effectiveness [15, 16].

An alternative approach for sharing experiential knowledge is the casebook, referring to a compilation of narrative accounts of QI experiences. For example, *Stories From the Floor* described multiple QI strategies employed in an overarching initiative to improve pain practices at one Canadian paediatric hospital [17]. Each chapter included a brief description of the setting and context, followed by content organized in a series of questions: Who was involved, what needed to change, what was done, what worked and why, what did not work and why, what was the impact, and what was learned? *Translating Knowledge Into Action* described QI strategies implemented to improve care across community, primary and acute care settings in England’s Yorkshire and Humber Collaboration for Leadership in Applied Health Research and Care [18]. Each chapter briefly described the context and project aims, approaches used, testimonials and project outcomes, and included a balance of text, infographics and tools. Such casebooks allow others to learn from a range of QI strategies, and choose and tailor strategies to their own context. They may be easy to develop, and less costly and complex to implement compared with QI collaboratives or coaches. However, it is unclear how widely casebooks are used, and the ideal characteristics and impact of casebooks are unknown. The overall aim of this research was to review published research to generate insight on the use and impact of casebooks as a means of sharing essential experiential QI knowledge and advice. The objectives were to describe QI casebook prevalence, development, characteristics and impact.

## Methods

### Approach

We conducted a scoping review based on currently recommended methods for scoping, searching, screening, data extraction and data analysis; and complied with a reporting checklist specific to scoping reviews [19–21]. We chose a scoping review over other types of syntheses because it is characterized by the inclusion of a range of study designs and processes or outcomes, which facilitates exploration of literature in a given field, reveals the nature of existing knowledge, and identifies issues requiring further primary study [22]. Similar in rigour to a systematic review, scoping reviews do not apply or generate theory, nor do they assess the methodological quality of included studies. We did not require research ethics board approval as data were publicly available. We did not publish a protocol.

### Scoping

To scope, or become familiar with the literature on this topic, we conducted an exploratory search in MEDLINE using keywords: [casebook OR case book]. The purpose was to assess examples of potentially relevant studies, and use that information to generate eligibility criteria based on the PICO framework (participants, issue, comparisons, outcomes), and develop a more elaborate search strategy. NA and ARG screened and discussed titles and abstracts, and together drafted eligibility criteria.

### Eligibility

We included studies in which participants were developers, or actual or potential users of casebooks including patients, family members or care partners with any disease/condition or healthcare issues, or clinicians in any setting of care, managers or executives in healthcare organizations, healthcare policy-makers, scientists or researchers.

With respect to issue, we defined a casebook as an educational tool designed to provide examples of implementing a strategy (practice, process, intervention, tool, etc.) aimed at improving the quality or safety of healthcare programmes or services. Based on aforementioned examples [17, 18], we characterized a casebook by one or more accounts of how individuals or organizations implemented one or more QI strategies along with additional considerations or instructions. In studies evaluating casebooks, they could be in print or electronic format, and delivered alone or in combination with one or more interventions (multifaceted). To be comprehensive and cast a wide net, we searched for casebooks alternatively labelled by authors as implementation guide, handbook, guidance document, resource, strategy(ies), framework, idea book, manual or reference book.

Regarding comparisons, eligible studies described or evaluated one or more casebooks, or cognitive or behavioural impacts after the introduction of a casebook; or compared cognitive or behavioural impacts before and after casebook introduction, or between groups exposed to different interventions with or without a casebook alone or combined in a multifaceted intervention. To capture this range of studies, we included English-language qualitative, quantitative or mixed/multiple-methods studies that developed or evaluated a casebook.

Outcomes included but were not limited to awareness or use of a casebook, determinants (enablers, barriers) of casebook use, or the impact of a casebook on individuals (e.g. knowledge, attitude, behaviour) or organizations (e.g. policies, culture change).

We excluded studies that focused on trainees, in non-healthcare contexts (e.g. education or sports), where the casebook was developed by a for-profit organization and not publicly available, based on casebooks comprised of simulated cases or expert opinion rather than real-world examples, that mention a casebook with no further description, or did not develop or evaluate a casebook but conclude by recommending a casebook. We also excluded publications in the form of clinical cases or case series, editorials, commentaries or abstracts.

### Searching

ARG, who has medical librarian training, developed a comprehensive search strategy (Additional file 1) that followed the Peer Review of Electronic Search Strategy reporting guidelines [23]. The search strategy combined Medical Subject Headings with a range of keywords in various combinations to identify relevant literature regardless of label used by authors to refer to a casebook. We searched MEDLINE, EMBASE, CINAHL and Scopus from inception to 4 February 2021, when searches were last updated.

### Screening

To pilot test screening, NA and ARG independently screened the same 20 titles and abstracts against eligibility criteria, discussed the results, resolved discrepancies, and refined eligibility criteria. Thereafter, NA screened the remaining titles and abstracts, consulted ARG regarding uncertainties, and retrieved the full text of potentially eligible items.

### Data extraction and analysis

We developed a data extraction form to collect information on study characteristics (author, publication year, objective, research design, results) and casebook definitions, development, characteristics, use and impact. We described characteristics using the Workgroup for Intervention Development and Evaluation Research (WIDER) framework (content, format, delivery, participants, personnel) [24]. As a pilot test, NA and ARG independently extracted data from two articles, discussed results, resolved discrepancies, and refined the data extraction approach. Thereafter, NA extracted data from remaining studies, and consulted ARG about uncertainties. ARG reviewed all extracted data. We used summary statistics, tables and text to report study characteristics, and casebook definitions, development, characteristics and impact.

## Results

### Search results

**Fig. 1:**
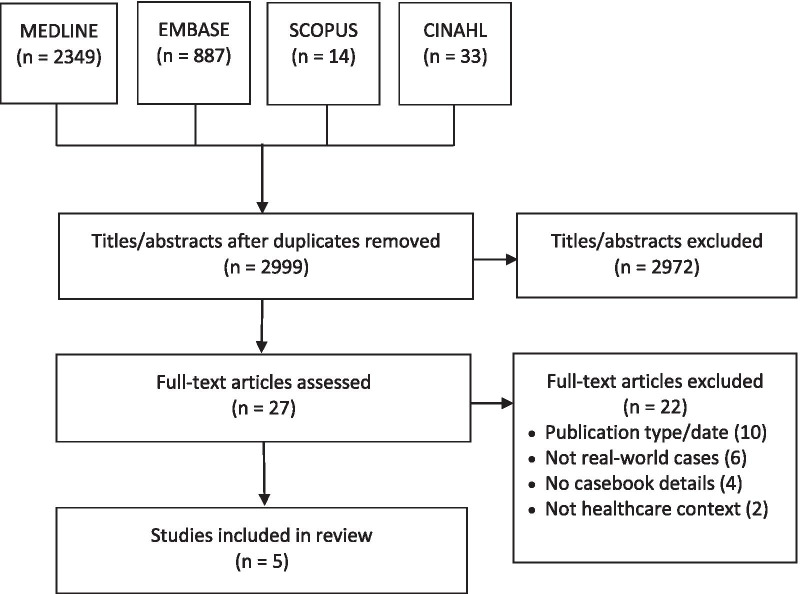
PRISMA (Preferred Reporting Items for Systematic Reviews and Meta-Analyses) diagram. Flow chart of studies screened and included

We identified 2999 unique articles across all databases searched, excluded 2972 titles/abstracts, screened 27 full-text articles and excluded 22 due to the following: publication type (10), not based on real-world examples (6), mentioned or recommended a casebook with no further details (4), or the context was not healthcare (2). We included five articles for review (Fig. 1). Additional file 2 tabulates data extracted from included articles [25–29].

### Study characteristics

All articles were published in Canada from 2011 to 2020. The five articles described three studies: one study developed and evaluated one casebook on positive weight-related conversations with children and parents [25, 26]; another study developed and evaluated another casebook on coordination of cancer care between primary care providers and cancer specialists [27, 28]; and the third study developed a casebook to describe QI across the cancer control continuum [29]. Regarding research design, one study employed a before/after cohort study to assess casebook adoption and impact [25]. All other studies described casebook development and characteristics.

### Casebook definition

One study defined a casebook as a type of knowledge translation product that, through stories, provided information and shared the experiences, knowledge and work of others with the intent of fostering knowledge and behaviour [25, 26]. Another study defined a casebook as “in-the-field” examples of strategies to improve care [27, 28]. A third study by the same author group on a distinct casebook referred to the casebook as “in-the-field” QI projects to provide useful direction to groups and individuals who face similar problems and challenges, and further specified project eligibility criteria: specific clinical problem, deliberate and organized effort was developed and implemented to address the problem, and the project was evaluated [29].

Overall, casebooks in included studies were meant to disseminate or share knowledge by describing real-world experiences of developing and implementing QI strategies such that others could learn from, emulate or adapt those strategies.

### Casebook development

One study conducted a scoping review to identify strategies, reviewed that evidence, and collected additional information through structured focus groups and interviews with children and parents; held two workshops with children, family and clinicians to refine content and key messages based on their experiences and preferences, and asked workshop participants to review the casebook [25, 26]. Two studies contacted leaders and experts to nominate and briefly describe projects, collected more detail through interviews with project representatives, or by searching for additional information in publications or on the Internet, then asked project representatives to review and refine their profile [27–29].

Overall, while reported details were few, casebook development was a multistep process where developers identified projects, collected project information via publications and/or from project representatives, prepared project descriptions and other content, and asked representatives to review and improve those descriptions.

### Casebook characteristics

Table 1 summarizes the characteristics of casebooks in included studies. Content differed by level of application: in one study, the casebook provided insight to improve communication between children and families and clinicians [25, 26]; the casebooks in the other two studies aimed to share improvements in cancer care coordination through organizational or regional strategies [27–29]. With respect to format, in one study, the casebook was organized by topic, and included case studies and anecdotes plus strategies and planning or implementation tools for each topic [25, 26]. Casebooks in the other two studies were organized by project, but included similar content such as strategies, tools and solutions for possible barriers [27–29]. All three casebooks were made available as electronic documents that could be downloaded and printed. One study delivered their casebook to users through a 2-hour in-person or virtual educational workshop including didactic, interactive, simulation and reflective learning based on casebook content [25, 26]. In one study, target users participated in developing casebook content through multiple, successive stages [25, 26]. The other two studies collected information from project leads and asked them to review the final product [27–29]. In one study, casebook developers included an interdisciplinary research team and a family member [25, 26]; casebooks in the other two studies were developed by study authors [27–29].

**Table 1:** Casebook characteristics

Study	Content	Format	Delivery	Participants	Personnel
Bonder [25] 2020 Provvidenza [26] 2019	The casebook addressed five topics: 1. Who should participate in weight-related discussions? 2. When and how should the topic be raised? 3. What should healthcare professionals say? 4. How can healthcare professionals enhance family engagement? 5. What techniques have been recommended?	Each of the five sections included checklists, evidence summaries, case studies, sentence starters, simulations, practice tips, illuminating anecdotes	Print/electronic document shared via 2-hour in-person or virtual educational workshop including didactic, interactive, simulation and reflective learning based on casebook content	Evaluation: 7 physicians, 1 nurse practitioner and 1 behavioural therapist Development: Interviews or focus groups with 18 children aged 7 to 18 years and 21 parents; 2 workshops with 22 youth, family members, clinicians and researchers; final review by 14 workshop participants and content experts	The research team included a dietitian, endocrinologist, child neurologist, nurse, weight communication expert, knowledge translation expert and a family member
Tomasone [27] 2017 Brouwers [28] 2016	Strategies to improve coordination of cancer care between primary care providers and oncology specialists for the diagnosis, treatment or follow-up/survivorship care of persons with breast or colorectal cancer	Cases included a brief outline of the project, stage of care, type of strategy, enablers and barriers, and evaluation of use or impact	Print/electronic document	Evaluation: None Development: Interviews with 24 project leads to collect information	Researchers
Brouwers [29] 2011	Quality improvement projects to improve problems related to the diagnosis or treatment of breast, gastrointestinal and genitourinary cancer at any stage	Cases included a brief outline of the project, stage of care, problem, type of strategy, enablers and barriers, tips and tools, and evaluation of use or impact	Print/electronic document	Evaluation: None Development: Leads of 19 projects reviewed their profile developed by the researchers	Researchers

Overall, while reported details of casebook characteristics were sparse, casebooks differed in whether they were organized by healthcare topic or by case, but included similar information such as case description, one or more strategies, and tips, tools and solutions to barriers for planning, developing and implementing those strategies.

### Casebook evaluation

Two studies summarized details of projects included in casebooks, but did not evaluate casebook use or impact [27–29]. One study reported that the casebook was downloaded 2497 times across five countries over a 1-year period [26], and evaluated its acceptability and impact among participants of two workshops used to disseminate the casebook [25]. All participants in both groups agreed that the workshop gave them a clear understanding of casebook content, helped them easily navigate the casebook and know when to use it, and perceived that it improved their self-efficacy in having weight-related conversations. Measured self-efficacy in having weight-related conversations increased on all variables from before the workshop to 2 months afterwards, and four (44%) said they used language and terminology from the casebook in weight-related conversations.

### Casebook template

We summarized findings to create a template of casebook characteristics that can be further developed through use by others for casebook development or evaluation (Table 2).

**Table 2:** Template of casebook characteristics

Domain	Category	Options
Development	Approach	Interdisciplinary participants Collaborative Iterative steps
	Identify strategies	Search for QI initiatives or strategies: - Indexed databases of published research - Online Consult experts or leaders - Survey - Interviews/focus groups
	Gather detail	Analyse the content of published studies or online reports; Conduct surveys, interviews or focus groups with QI project representatives or other stakeholders (e.g. patients, family)
	Review cases	Ask QI project representatives or others for feedback to refine content and format
Characteristics	Content	Varies by QI topic
	Level of application	Individual Organizational Regional
	Scope	One or more QI strategies
	Format	Organize cases by: - QI topic - QI strategies Includes text, graphics, tools
	Case	Context (setting, people involved) Problem QI strategy details Anecdotes, quotes or testimonials Enablers, barriers Solutions, tips
	Delivery	Print or electronic Shared online or by email Disseminated via educational workshop
Evaluation	Fidelity	Downloads, self-reported use
	Satisfaction	Acceptability, user experiences
	Cognitive impact	Knowledge Attitudes Self-efficacy
	Behavioural impact	Adoption of practice (self-reported, objectively measured)
	Organizational impact	Teamwork Culture Efficiency
	Clinical impact	Safety measures Clinical outcomes

## Discussion

We conducted a scoping review to assess QI casebook prevalence, development, characteristics and impact. With respect to prevalence, only three studies reported in five articles were eligible. Regarding development, the creation of casebooks was a multistep process of identifying relevant projects followed by iterative collection and refinement of details with project representatives. Projects or initiatives included in casebooks featured common elements, often communicated with a blend of text, graphics and tools. Regarding impact, only one study evaluated outcomes and found that the casebook was acceptable to users and used in practice [25].

The paucity of research on casebooks was somewhat surprising given that numerous examples exist in the “grey” literature [17, 18]. Given uncertainty about the effectiveness of QI strategies [4–6] or approaches to support QI [12–16], this research addressed a gap in knowledge on how to share wisdom and advice about QI generated by multiple initiatives conveniently compiled in a casebook, enabling others to learn from and apply those experiences in their own context. Definitions and descriptions of the intent of casebooks included in eligible studies support the idea that stories about “in-the-field” experiences convey experiential knowledge, which appears to be an essential ingredient, on its own, or possibly supplementary to other knowledge-sharing strategies that support QI such as QI collaboratives or coaching [9–11]. The fundamental role of experiential knowledge in healthcare was established by what is considered a “landmark” study, which showed that clinicians rarely used published research, guidelines or other forms of codified knowledge, and instead relied on “mindlines”, defined as collectively reinforced, internalized experiential guidelines, informed mainly through interactions with colleagues and opinion leaders, and by other sources of largely experiential knowledge [30]. A casebook may well be another form of codified knowledge that clinicians or other healthcare professionals ignore. Alternatively, given that mindlines are collectively reinforced, perhaps experiential knowledge (versus clinical evidence) shared via a casebook might contribute to the formation of mindlines.

This research generated insight on how to develop casebooks. In all cases, development was a multistep process where developers identified relevant projects through nomination or searching, collected project information via publications and/or from project representatives, prepared project descriptions and other content, and asked representatives to review and improve those descriptions. Given growing interest in casebooks, others have begun to develop systematic methods for creating casebooks. For example, recognizing that compilations of “real-world” initiatives represent an important source of information for QI efforts, D’Urzo et al. developed an approach for identifying relevant cases through peer-reviewed literature databases, grey literature databases, customized Google searches, targeted websites and consultation with content experts, and illustrated this approach by using it to search for community-based physical activity programmes for persons with physical disabilities [31]. Clearly, coproduction or collaboration among interdisciplinary stakeholders is inherent in casebook development and likely leads to greater relevance and use of casebooks. Given broad attention to stakeholder engagement in QI and in research, considerable guidance is available on how to undertake and optimize stakeholder engagement [32–35].

This research also generated insight on the content and format of QI casebooks. While casebooks varied in QI topic, level of application (individual, organizational, regional) and scope (one or more QI strategies), cases featured common elements: brief description of setting or context, details about one or more QI strategies, impacts or outcomes achieved, and additional insight (tips, tools, solutions to barriers) for planning, developing and implementing those strategies. By having documented these characteristics in a baseline template, future casebook developers and researchers can more consistently create and evaluate casebooks, and in so doing, build on this template, ultimately leading to a more definitive guide on how to develop casebooks.

The strengths of this study include use of rigorous scoping review methods [19, 20, 22] and compliance with standards for the conduct and reporting of scoping reviews and search strategies [21, 23]. We searched the most relevant databases of medical literature from inception. To organize findings, we mapped casebook characteristics to the established WIDER framework for reporting interventions [24]. Several limitations must also be noted. Our search was limited to English language studies, so we may not have included relevant studies published in other languages. The search strategy may not have identified all relevant studies, or our screening criteria may have been too stringent. The included studies were few, and provided limited and anecdotal details. All studies were published in Canada, so the context of a casebook may not be widely relevant. However, QI is practised worldwide, and several QI casebooks that are available online were developed in other countries [18, 36, 37]. In this study, we included only casebooks published in peer-reviewed literature for two reasons: one, we wished to first assess empirical work on casebook impacts, and two, we anticipated finding more studies than we did and wished to establish a feasible scope for work required.

While few studies have evaluated casebooks, many examples are available online [17, 18, 36–38]. In the future, we will build on this study by conducting a grey literature search for QI casebooks and analysing their content to elaborate the baseline casebook template reported here. Ongoing research is needed to generate insight on optimal methods for developing casebooks. Further research is also needed to rigorously evaluate the impact of casebooks alone or in combination with other interventions that support QI on a range of outcomes, including the implementation and fidelity of QI strategies, and the impact of those strategies on professional practice, patient experience and other patient-important outcomes, and clinical outcomes associated with practice improvements.

## Conclusions

Casebooks are one mechanism for sharing experiential knowledge essential to planning and implementing QI strategies, and many examples are available online. This scoping review revealed a paucity of research on casebooks. However, synthesis of five articles pertaining to three casebooks revealed practical knowledge upon which future research can build. Casebook development is a multistep, iterative, interdisciplinary process that engages stakeholders in identifying, creating and reviewing content. Casebooks may differ in QI topic, level of application or scope, but cases featured common elements: setting or context, QI strategy details, impacts or outcomes achieved, and additional insight (tips, tools, solutions to barriers) for planning, developing and implementing those strategies. Cases were described with a blend of text, graphics and tools. Characteristics were summarized in a casebook template, which others can use as the basis for developing or evaluating casebooks. One study demonstrated that a casebook can improve clinical care. Future research is needed to optimize methods for developing casebooks and to evaluate their impact.

## Supplementary Information

**Additional file 1.** MEDLINE search strategy. Table showing search terms and combinations used to search the MEDLINE database for published research.

**Additional file 2.** Data extracted from included studies. Table of data on study characteristics, and casebook development, characteristics and impact extracted from included studies.

## Data Availability

All data generated or analysed during this study are included in this published article and its supplementary information files.

## Abbreviation

QI: Quality improvement
