# Supplementary material for: Development, characteristics and impact of quality improvement casebooks: a scoping review
Source: Health Res Policy Syst. 2021 Sep 8;19:123. doi: 10.1186/s12961-021-00777-z (PMC8425030; doi:10.1186/s12961-021-00777-z)
Supplement: Supplementary file 1 — Additional file 1. MEDLINE search strategy. Table showing search terms and combinations used to search the MEDLINE database for published research. [file 12961_2021_777_MOESM1_ESM.docx]

Additional File 1. MEDLINE search strategy

| **#** | **Search Statement** | **Results** |
| --- | --- | --- |
| 1 | (case book or casebook or case-book).mp. | 500 |
| 2 | (implement* adj4 guid*).mp. | 13287 |
| 3 | (implement* adj4 manual).mp. | 249 |
| 4 | (implement* adj4 instruct*).mp. | 620 |
| 5 | 2 or 3 or 4 | 14117 |
| 6 | knowledge transfer.mp. | 1779 |
| 7 | knowledge translation.mp. | 2915 |
| 8 | communication/ | 81897 |
| 9 | health communication/ | 2114 |
| 10 | diffusion of innovation/ | 17380 |
| 11 | translational medical research/ | 10216 |
| 12 | information dissemination/ | 16093 |
| 13 | implementation science/ | 271 |
| 14 | problem-based learning/ | 8072 |
| 15 | health education/ | 59797 |
| 16 | health promotion/ | 71987 |
| 17 | patient education as topic/ | 83853 |
| 18 | program development/ | 28437 |
| 19 | quality of healthcare/ | 71437 |
| 20 | quality assurance, healthcare/ | 55474 |
| 21 | health plan implementation/ | 5688 |
| 22 | organizational innovation/ | 24121 |
| 23 | or/6-22 | 490246 |
| 24 | 5 and 23 | 2516 |
| 25 | 1 or 24 | 3016 |
| 26 | limit 25 to english language | 2811 |
| 27 | limit 26 to (autobiography or biography or case reports or classical article or clinical trial, all or comment or editorial or historical article or interview or lecture or letter or news or personal narrative) | 563 |
| 28 | 26 not 27 | 2248 |
| 29 | from 28 keep 1-1000 | 1000 |
| 30 | from 28 keep 1001-2248 | 1248 |
