# Supplementary material for: Development, characteristics and impact of quality improvement casebooks: a scoping review
Source: Health Res Policy Syst. 2021 Sep 8;19:123. doi: 10.1186/s12961-021-00777-z (PMC8425030; doi:10.1186/s12961-021-00777-z)
Supplement: Supplementary file 2 — Additional file 2. Data extracted from included studies. Table of data on study characteristics, and casebook development, characteristics and impact extracted from included studies. [file 12961_2021_777_MOESM2_ESM.docx]

Additional File 2. Data extracted from included studies

| Study Country | Objective | Casebook definition/description | Research Design | Results |
| --- | --- | --- | --- | --- |
| Bonder [25]  2020  Canada  SEE Provvidenza 2019 for development of this casebook | Explore the acceptability and adoption of a casebook to help clinicians have positive weight-related conversations with children and parents | Casebooks are a type of knowledge translation product. Through stories, casebooks provide  information and share the experiences, knowledge, and work of others such as researchers and healthcare providers with the hope of fostering knowledge and behaviour | Before-after cohort study  Casebook introduced through a 2-hour in-person (n=5 from a pediatric teaching hospital) or online (n=4 from primary care settings across region) educational workshop comprised of didactic, interactive, simulation and reflective learning based on casebook content  Participants included 7 physicians, 1 nurse practitioner and 1 behavioural therapist (all women) | All participants in both groups agreed that the workshop gave them a clear understanding of casebook content, helped them easily navigate the casebook and know when to use it, and improved their self-efficacy in having weight-related conversations  Self-efficacy in having weight-related conversations increased on all variables from before to 2 months after the workshop  With respect to practice, 4 (44%) participants used the language and terminology from the casebook in their weight-related conversations. |
| Provvidenza [26]  2019  Canada | To develop a casebook that helps clinicians have positive weight-related conversations with children and parents | Casebooks use informal and narrative approaches to share research evidence and the experiences of key stakeholders. The goal is to provide knowledge and raise awareness, with the hope of fostering changes in knowledge and practice | Scoping review of 32 studies published from 2005-2016 to identify evidence on best practices in weight-related communication  Review of that evidence with children aged 7 to 18 years (n=18) and their parents (n=21) recruited from two pediatric hospitals via focus groups and individual interviews  Two workshops involving 22 youth ambassadors, family members, and clinicians and researchers recruited from weight- management clinics, obesity organizations and a family leadership program who refined key messages and how to present that information  Final review of draft casebook by 14 individuals from the workshops and content experts | The casebook addressed 5 topics; each included checklists, evidence summaries, case studies, sentence starters and simulations:   1. Who should participate in weight‐related discussions? 2. When and how should the topic of weight be broached? 3. What terminology should be used? 4. How can HCPs enhance family engagement in weight‐related discussions? 5. What specific communication techniques have been recommended?   The casebook was downloaded 2,497 times across 5 countries from November 2017 to October 2018 |
| Tomasone [27] 2017 and Brouwers [28] 2016  *this casebook is distinct from Brouwers 2011 | To develop and describe a casebook of projects that supported breast and colorectal cancer care coordination between primary care providers and oncology specialists | “In-the-field” examples of strategies to improve coordination of cancer care between primary care providers and oncology specialists for the diagnosis, treatment or follow-up/survivorship care of persons with breast or colorectal cancer | Cancer system leaders across Canada were contacted to self-nominate or nominate others and provide a brief summary of projects that met eligibility criteria. The research team reviewed 159 nominations, interviewed representatives of 24 selected projects to collect more details, and asked project leads to review profiles.  Describe casebook:  Cancer diagnosis, stage of cancer care continuum, jurisdiction, primary care engagement, strategy employed | Projects focused on needs of patients undergoing treatment (15) or on follow-up care or survivorship (11)  Primary care engagement: 7 low, 11 moderate and 6 high; those engaged provided advice and support for project implementation and some acted as champions to promote the project  Strategies included nurse navigation, multidisciplinary teams, electronic communication or information systems, or primary care provider education  Barriers: lack of care standardization across proximate jurisdictions and incompatibility among electronic communication or information systems.  Enablers: leadership support, repurposing existing resources, financial support, and a motivated and skilled project team  11 projects were formally evaluated; outcomes included patient and provider satisfaction, improvements in clinical efficiency, and resource utilization savings |
| Brouwers [29]  2011  Canada | To develop and describe a casebook of cancer-related quality improvement projects | Quality improvement activities aim to solve a specific problem in a specific context. Quality improvement initiatives can be valuable by providing useful direction to groups and individuals who face similar problems and challenges in other contexts.  Eligibility: “In-the-field” projects had to demonstrate that a specific cancer control problem was identified; that a deliberate and organized effort was developed and implemented to address the identified problem; and that an evaluation—formal or informal—was used to assess the effort. | Develop casebook:  Cancer system leaders across Canada were contacted to self-nominate or nominate others and provide a brief summary of projects that met eligibility criteria. The research team reviewed 30 nominated projects and selected 19 that were further advanced (had been implemented and/or evaluated) and represented the continuum of cancer care. They collected additional information about each project from publications and the Internet, then asked project leads to review the profile.  Describe casebook:  3 research team members independently extracted information and then compared results to achieve consensus on: problem, barriers, solutions, model/framework, evaluation and impact, plus stage of cancer continuum, cancer diagnosis, jurisdiction and scope. | Of the 19 projects, most pertained to breast cancer (10), treatment (11), were regional (19) and at the level of the organization (22).  5 projects used a model or framework to guide evaluation  16 reported formal evaluation, most often surveys to assess behaviour or clinical outcomes such as screening rates, wait times, and office or hospital visits  Common barriers: financial and administrative challenges  Common enablers: collaborative teamwork, project planning and use of a systematic process, and support including leadership endorsement, funding and engaged people |
